# Antimicrobial Actions and Applications of Chitosan

**DOI:** 10.3390/polym13060904

**Published:** 2021-03-15

**Authors:** Cai-Ling Ke, Fu-Sheng Deng, Chih-Yu Chuang, Ching-Hsuan Lin

**Affiliations:** Department of Biochemical Science and Technology, College of Life Science, National Taiwan University, Taipei 10617, Taiwan; d04b22004@ntu.edu.tw (C.-L.K.); r08b22027@ntu.edu.tw (F.-S.D.); r09b22012@ntu.edu.tw (C.-Y.C.)

**Keywords:** chitosan, antimicrobial activity, physicochemical characteristics, nanoparticles, films

## Abstract

Chitosan is a naturally originating product that can be applied in many areas due to its biocompatibility, biodegradability, and nontoxic properties. The broad-spectrum antimicrobial activity of chitosan offers great commercial potential for this product. Nevertheless, the antimicrobial activity of chitosan varies, because this activity is associated with its physicochemical characteristics and depends on the type of microorganism. In this review article, the fundamental properties, modes of antimicrobial action, and antimicrobial effects-related factors of chitosan are discussed. We further summarize how microorganisms genetically respond to chitosan. Finally, applications of chitosan-based biomaterials, such as nanoparticles and films, in combination with current clinical antibiotics or antifungal drugs, are also addressed.

## 1. Introduction

Chitin (β-(1–4)-poly-N-acetyl-D-glucosamine) is the second most abundant polysaccharide distributed in nature [1]. Chitin can be easily found in a variety of organisms, particularly in the exoskeletons of insects, lobsters, shrimp, and crabs [1,2]. In addition, chitin (poly-(β-1→4)-2-amino-2-deoxy-d-glucopyranose) is the major source of chitosan, which is obtained by removing the acetyl group (CH_3_-CO) from chitin [1,2,3,4]. In addition to enzymatic processes, the preparation of chitosan mainly relies on chemical processes to remove the minerals and proteins present in shellfish [1,2,3,4,5,6,7]. Briefly, hydrochloric acid (HCl) is often utilized as the preferred reagent during the demineralization process [4,5]. In the second step, sodium hydroxide (NaOH) is used at 65–100 °C for 0.5–72 h for deproteinization and deacetylation [4,5]. The processes of demineralization and deproteinization profoundly affect the molecular weights (MWs) and distribution of deacetylated chitosan [4,5]. For example, treatment for a long period of time and incubation at high temperatures during deproteinization often produces low molecular weight and highly deacetylated chitosan [1,2,3,4,5,6,7].

Due to its several unique properties, including biodegradability, biocompatibility, and low toxicity, chitosan has been extensively investigated for applications in many fields. For example, chitosan has been used as a flocking agent in water treatment [8,9,10,11,12,13,14,15,16,17,18,19,20,21,22], an elicitor to activate plant defenses [23,24,25,26,27,28,29,30,31,32,33,34,35,36,37], a supplement during food preservation and in food additives [38,39,40,41,42,43,44], a dehydrating agent in cosmetics [45,46,47,48,49,50,51], and a drug delivery carrier [52,53,54,55,56,57,58,59,60,61,62,63,64,65,66,67,68,69,70,71] and a hydrogel film in pharmaceutical areas [59,70,72,73,74,75,76,77,78,79,80]. Furthermore, the broad antimicrobial activity of chitosan against bacteria and fungi has been reported in many articles [72,81,82,83,84,85,86,87,88,89,90,91,92,93,94,95,96,97,98,99,100,101,102,103,104,105,106]. However, the effectiveness of the antimicrobial activity of chitosan is highly dependent on the type of target microorganism [29,36,84,88,90,103,107]. Furthermore, the mechanisms of the antimicrobial activity of chitosan are associated with its physicochemical properties [4,37,43,90,103,108,109,110,111,112]. Thus, this review article highlights the antimicrobial properties of chitosan, the factors that influence its antimicrobial activity, how bacteria and fungi respond to chitosan, and what regulators are involved in this antimicrobial response. Additionally, future perspectives on chitosan, in addition to problems in further applications, are addressed.

## 2. Antimicrobial Actions of Chitosan

The mechanisms of action of chitosan against bacteria and fungi have been investigated and reported in many articles [72,81,82,83,84,85,86,87,88,89,90,91,92,93,94,95,96,97,98,99,100,101,102,103,104,105,106]. Although the antimicrobial properties of chitosan are highly associated with its structure, physicochemical characteristics, and environmental conditions, in addition to the reactive hydroxyl groups at the C-3 and C-6 positions [4,37,39,43,90,103,105,108,109,110,111,112,113,114,115,116,117,118,119,120], the mode of action of chitosan against microbes can be classified as extracellular effects, intracellular effects, or both based on the targeting site of the antimicrobial effects [36,68,90,103,107,121]. Because high-MW chitosan is generally unable to penetrate the cell wall and cell membrane, its potential antimicrobial effects involved acting as a chelator of essential metals, preventing nutrients from being taken up from cells extracellularly, and altering cell permeability [68,84,90]. However, low-MW chitosan not only has extracellular antimicrobial activity but also intracellular antimicrobial activity, thereby affecting RNA, protein synthesis, and mitochondrial function [68,84,90,122,123]. Furthermore, the mode of antimicrobial action of chitosan is highly dependent on the type of targeted microorganism.

### 2.1. Antimicrobial Activity against Bacteria

**Figure 1:**
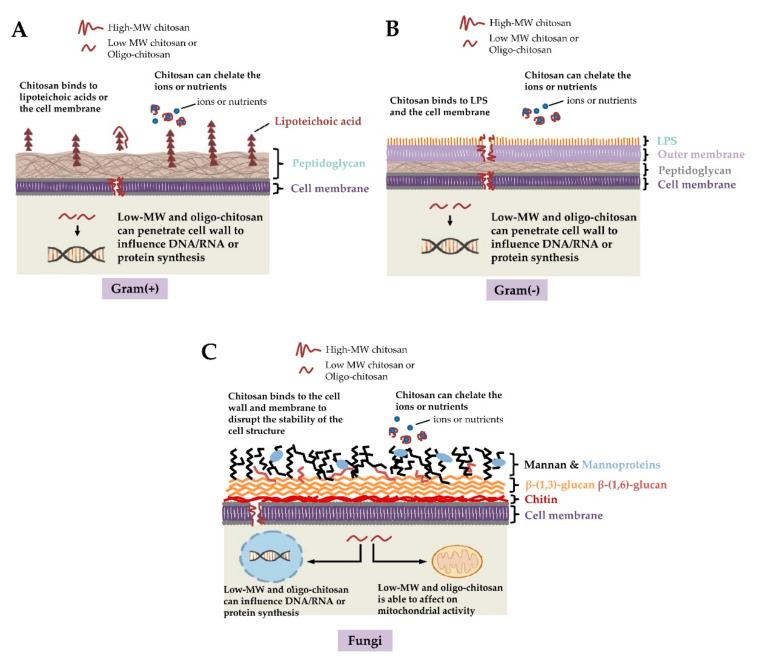
Potential antimicrobial actions of chitosan against (**A**) gram-positive bacteria, (**B**) gram-negative bacteria, and (**C**) fungi. Given the negative charges of teichoic acids in gram-positive bacteria, lipopolysaccharide (LPS) in gram-negative bacteria and the phosphorylated mannosyl side in fungi, electrostatic interactions occur between the positively charged chitosan and the cell surface of the microorganism. Furthermore, chitosan chelates the environmental ions and nutrients required for bacterial survival. Low-molecular weight (MW) chitosan and oligo-chitosan might affect DNA/RNA or protein synthesis after passing through the cell wall and cell membrane into the cytoplasm. Additionally, low-MW chitosan and oligo-chitosan inhibit mitochondrial function and ATP production.

Gram-positive and gram-negative bacteria exhibit remarkable differences in their cell wall structure, in which gram-positive bacteria have thicker peptidoglycans and gram-negative bacteria are enriched in lipopolysaccharide (LPS) [124,125,126,127]. Differences in the cell surface structure of these types of bacteria also lead to distinct susceptibilities to chitosan. For example, gram-negative bacteria present a more negative charge than gram-positive bacteria because LPS is often attached to phosphorylated groups [128,129]. More negatively charged cell surfaces allow the binding of cationic chitosan to phospholipids when the environmental pH is below 6.5 [2,68,90,103,107,121,130,131]. The potential antibacterial action of chitosan is shown in Figure 1A,B. It has been suggested that gram-negative bacteria could be more susceptible to chitosan than gram-positive bacteria [114,132,133,134], but some studies have shown that gram-positive bacteria are more sensitive to chitosan [88].

Teichoic acids in gram-positive bacteria are also negatively charged due to the presence of phosphate groups in their structure [124,125]. However, deletion of the teichoic acid biosynthesis pathway in *Staphylococcus aureus* resulted in increased resistance to chitosan [107], indicating that the mode of action of chitosan is more complex than simple electrostatic interactions. In addition, unlike gram-negative bacteria, gram-positive bacteria have a thick cell wall, which might prevent chitosan from binding directly to the cell membrane. However, some chitosan oligomers (<5 kDa) penetrate the cell wall and influence DNA/RNA or protein synthesis [68,84,90,122,123]. Interestingly, reports have demonstrated that chitosan (≤50 kDa) can pass through the cell wall and inhibit DNA transcription [68]. Thus, although the molecular size of chitosan plays an important role in targeting, the structure rather than the MW of chitosan also determines its extracellular, intracellular, or both extracellular and intracellular antibacterial activity.

### 2.2. Antimicrobial Activity Against Fungi

Chitosan has been shown to have fungicidal effects on several fungal pathogens in plants and humans [82,83,108,135,136,137,138,139,140,141,142,143,144,145,146,147]. Its antifungal properties are mainly related to the interaction of chitosan with the cell wall or cell membrane. Nevertheless, the minimum inhibitory concentrations (MICs) of chitosan against fungi vary and are highly associated with the MW and degree of deacetylation (DDA) of chitosan, solvent pH, and the type of fungus being targeted [2,90,107,136,140,144,148,149]. Furthermore, the unsaturated fatty acid contents on the cell membrane might be positively correlated with chitosan susceptibility [150] because a higher content of unsaturated fatty acids exhibits better membrane fluidity, leading to a more negative charge on the cell membrane [151]. For example, the opposite characteristics of chitosan-sensitive and chitosan-resistant *Neurospora crassa* strains are related to the content of unsaturated fatty acids on the cell membranes [150]. These data may account for, at least in part, why *Candida albicans*, *Candida tropicalis*, and other *Candida* species have remarkable differences in susceptibility to the same chitosan [83,152]. Indeed, *C. tropicalis* exhibited an increase in susceptibility of more than 1,000-fold to certain chitosans compared with *C. albicans* [83,152]. Similarly, in addition to its extracellular antifungal effects, low-MW chitosan is able to penetrate the cell wall and cell surface, leading to the inhibition of DNA/RNA and protein synthesis [39,68,82,91,122,125,132]. Interestingly, a previous report has further suggested that chitosan affects mitochondrial activity [123]. The mode of action of chitosan against fungi is shown in Figure 1C.

## 3. Factors Influencing the Antimicrobial Activity of Chitosan

### 3.1. pH

A major antimicrobial effect of chitosan is electrostatic interactions between this cationic molecule and negatively charged cell walls [72,81,82,83,84,85,86,87,88,89,90,91,92,93,94,95,96,97,98,99,100,101,102,103,104,105,106]. The pKa values of the amino groups of chitosan are 6.3–6.5, indicating that it is insoluble in alkaline solutions, organic solvents, and water when the pH is higher than 6.5 [36,68,90,103,107,121]. Additionally, the solubility increases with decreasing solution pH, which leads to an increase in the positive charge on the –NH_3_ groups of chitosan and stronger antimicrobial activity [36,68,90,103,107,121]. In fact, a large number of articles have demonstrated that chitosan exhibits excellent antimicrobial activity under acidic conditions, as summarized in several review articles [36,68,90,103,107,121].

### 3.2. Molecular Weight

The molecular weight of chitosan determines whether it penetrates the cell surface to exert intracellular antimicrobial activity. Furthermore, the abundance of polysaccharides and a few proteins that compose the complex layers of the cell wall in both bacteria and fungi not only play important roles in pathogenesis, biotic surface adhesion, and abiotic surface adhesion, and induction of the immune response, but also offer mechanical strength and a barrier from the environment [124,153,154,155]. In fact, the rigid cell wall transports molecules across the outer layer barrier via several delicate mechanisms or by simple diffusion [156,157,158], and the cell wall porosity and pore size determine whether a compound or molecule passes through the bacterial or fungal cell wall [156,157,158]. The pore sizes vary between different bacteria and fungi, with a range of 2–4 nm up to 8 nm [154,159,160,161,162,163]. For example, the pore sizes determined by fluorescein-labeled dextran are 2.06 and 2.12 nm in *Escherichia coli* and *Bacillus subtilis*, respectively, whereas *Pseudomonas aeruginosa* exhibits larger pores of 13 ± 5 nm [159,160,161,162]. Additionally, it has been proposed that the cell wall pore sizes in *Saccharomyces cerevisiae*, *C. albicans* and *Cryptococcus neoformans* are approximately 5.8 nm [160,163]. Based on pore size, only ~5 kDa (minimum radius: 1.1 nm) globular molecules or proteins can penetrate most bacterial cell walls, and 50 kDa (minimum radius: 2.4 nm) spherical molecules or proteins should be able to pass through fungal cell walls [164]. However, the hydration state influences sphere size, and the hydrodynamic radii of proteins are usually larger. For example, the radii of beef pancreas ribonuclease A (14 kDa), beef pancreas chymotrypsinogen A (25 kDa), and hen egg ovalbumin (43 kDa) are 1.05, 1.21 and 1.27 nm, respectively, in a nonhydrated state, but these radii increase to 1.64, 2.09 and 3.05 nm, respectively, in their hydrated state [164]. These data suggest that globular proteins with a molecular weight of 30 kDa or less can cross the microbial cell wall under physiological conditions. Similarly, chitosan has a diameter of ~1.1 nm in its linear extended form [165]; however, the hydrodynamic radius of hydrated chitosan (50–190 kDa) is 25.59 nm [107].

Reports have shown that oligo-chitosan (<5 kDa) can penetrate the cell wall, leading to intracellular antimicrobial activity [122]. Therefore, the question is how a ~50 kDa molecular weight chitosan might be able to penetrate the bacterial cell wall to inhibit DNA transcription [68,166]. Several possibilities might explain how larger chitosan molecules could enter cells: (1) Cell walls are dynamic structures that vary during replication, hyphal development, and age [124,153,154,155], and this flexibility may allow various molecules to pass through the cell wall. Indeed, a recent article has provided solid evidence of this phenomenon. Amphotericin B liposomes (AmBisomes) are liposomal delivery systems containing the antifungal drug amphotericin B [163]. Interestingly, AmBisome, which is 60–80 nm, is able to penetrate the cell walls of *C. albicans* and *C. neoformans* (pore size of 5.8 nm) [163]. These data suggest that the fungal cell wall is capable of remodeling and that the viscoelastic properties of the cell wall help larger molecules or compounds migrate through the outer layer. (2) Chitosan might affect cell wall porosity. Many reports have shown that environmental conditions and stresses profoundly influence cell wall porosity. For example, in *S. cerevisiae*, cell wall porosity increases after treatment with polyethylene glycol (PEG), dithiothreitol (DTT), or ethylenediaminetetraacetic acid (EDTA), whereas glucanase-soluble mannoproteins decrease the cell wall porosity of yeasts [160,167,168,169]. In addition to cell wall penetration, AmBisomes also transiently affect the cell wall porosity of *C. albicans* [163]. Therefore, chitosan may influence the cell wall pore sizes, but there is no evidence to support this hypothesis.

### 3.3. DDA

Given that the amino group (−NH_2_) of chitosan is the most important functional group, the DDA of chitosan influences the performance of chitosan in many applications [2,68,90,103,107,121,122,131]. The DDA of chitosan is highly associated with the preparation method, particularly the processing time and temperature used during chemical treatment [4,5]. Longer processing times and higher temperatures usually result in a high DDA [3,5,8,11,12]. Furthermore, chitosan with a high DDA has been shown to exhibit a more positive charge than chitosan with a low DDA in the same acidic environment [68,90,103,107,121,122,170,171]. Thus, chitosan with a high DDA has stronger electrostatic interactions with the microbial cell surface, which often results in better antimicrobial activity. Indeed, studies have shown that high DDAs of chitosan exhibit stronger antimicrobial activity against bacteria [170,171].

### 3.4. Derivatives

Although the antimicrobial activity of chitosan is affected by the pH, MW, and DDA, the physicochemical characteristics of the C2-NH_2_, C3-OH (secondary hydroxyl), and C6-OH (primary hydroxyl) functional groups of chitosan also significantly influence the antimicrobial properties. However, the antimicrobial effect of chitosan is observed only in acidic environments. Thus, due to the low solubility and lack of a positive charge at neutral pH, a large number of chitosan derivatives modified with amine (N-modified) and hydroxyl (O-modified) groups by acylation, carboxylation, alkylation, and quaternatization have been developed and investigated [43,58,97,119,120,132,133,142,172,173,174,175,176]. Furthermore, the secondary hydroxyl groups (C3-OH), which are difficult to modify, cause large steric hindrance. Herein, we mainly focus on N-modified chitosan derivatives, given that O-modified (C6-OH) chitosan derivatives are less studied. For example, many N-modified chitosan derivatives, such as acetylphenyl-thiosemicarbazone, N-benzyl and thymine-based chitosan, imino-chitosan, quaternary ammonium chitosan, and alkyl sulfonated derivatives, exhibited stronger antimicrobial activities against *Botrytis cinerea*, *E. coli*, *S. aureus*, *P. aeruginosa*, *Aspergillus nigger*, *Aspergillus fumigatus, Candida albicans*, *Colletotrichum gloeosporioides*, *Alternaria solani*, *Fusarium oxysporum* f. sp. *Vasinfectum*, *Pythium debaryanum*, and others [177,178,179,180,181,182]. Furthermore, the carboxymethyl chitosan-zinc complex of either N- or O-derivatives exhibits a better cidal effect against microbes [183]. Interestingly, both N- and O-modified (O-quaternary ammonium N-acyl thiourea) chitosan had greater bacterial growth inhibition than singly modified chitosan and pure chitosan [184].

## 4. Genetic Responses of Chitosan-Treated Bacteria and Fungi

### 4.1. Bacterial Responses

There have been few reports regarding the transcriptional responses of bacteria to chitosan. The microarray profile of chitosan-treated *S. aureus* SG511 showed that 84 genes and 82 genes were significantly upregulated and downregulated, respectively (Table 1) [107]. Chitosan treatment remarkably inhibited bacterial growth through downregulation of the genes involved in growth and metabolism, such as genes for RNA, protein, carbohydrate, amino acid, nucleotide, and lipid biosynthesis [107]. Furthermore, genetic profiles have suggested that chitosan impaired oxygen consumption and preferred anaerobic respiration [107].

A similar finding was found in *Bacillus cereus* after treatment with either type of polysaccharide or chitosans A and B, in which both chitosans significantly inhibited nitrogen, amino acid, and pyruvate metabolism, and gluconeogenesis (Table 1) [185]. Moreover, several genes involved in ion transport, particularly potassium transport, were upregulated [185]. However, *B. cereus* deficient in the genes required for potassium transport (the Kdp system) exhibited similar susceptibility to chitosan A and chitosan B compared to the wild-type strain [185], which may have been due to the Kdp system loss in *B. cereus* not being sufficient to block potassium uptake and enhance chitosan susceptibility.

### 4.2. Fungal Responses

Compared with bacteria, there have been relatively more studies showing how budding yeast and fungal pathogens respond to chitosan. In *S. cerevisiae*, as chitosan treatment time increased (15, 30, 60, 120, and 180 min), the number of up- and downregulated genes increased [186]. Functional analysis showed that genes involved in endoplasmic reticulum (ER), cell wall biogenesis, cell membrane biogenesis, and stress adaptation were significantly differentially expressed (Table 2). The ER is a key organelle that synthesizes lipids and membrane-associated proteins for the plasma membrane [186]. Furthermore, chitosan-treated *S. cerevisiae* exhibited less sensitivity to β-1,3-glucanase [186]. These data suggest that the cell wall and cell membrane are the targets of chitosan. A step further is the understanding of the transcription factor (TF) in *S. cerevisiae* that are involved in chitosan stress responses, which are Cin5p, Crz1, and Rlm1p. Cin5p is a basic leucine zipper (bZIP) that mediates drug resistance and stress tolerance. Crz1p, a calcineurin-responsive zinc finger, is required for calcium hemostasis and is activated in response to calcium. Rlm1p is a protein kinase involved in cell wall integrity [186]. These data suggest that chitosan may also have intracellular activity that influences gene expression.

Haploinsufficiency (HIP), homozygous deletion (Hop), and multicopy suppression (MSP) fitness assays of chitosan oligosaccharide (COS) combined with microarray analyses, showed that the response to COS is associated with the plasma membrane, respiration, and mitochondrial biogenesis, and 21 genes required for chitosan resistance in budding yeast were successfully identified (Table 2) [187]. Among these, overexpression of *ARL1*, which encodes a GTPase involved in the regulation of membrane organization and trafficking, resulted in reduced chitosan-induced membrane permeabilization [187]. Interestingly, *ARL1* overexpression did not confer resistance to salt and sugar stresses, and exhibited increased sensitivity to antifungal drugs, indicating that the chitosan-induced transcriptional response is distinct from those to antifungals and stresses.

*Aspergillus ochraceus* is one of the most abundant food-contaminating microorganisms due to mycotoxin production [188]. Chitosan treatment caused *A. ochraceus* to form abnormal hyphal branches and remarkably influenced cell wall and cell membrane architectures [189]. RNA sequencing analysis further demonstrated that chitosan inhibited genes involved in cell surface integrity and protein biosynthesis [189]. Chitosan upregulated phospholipase-related genes involved in membrane degradation and genes involved in steroid metabolism (Table 2) [189].

In *N. crassa,* chitosan treatment led to higher levels of intracellular reactive oxygen species (ROS), leading to plasma membrane permeabilization [190]. RNA sequencing analysis revealed that genes associated with mitochondrial function (4, 8, and 16 h treatment), peroxisome organization (4 h treatment), oxidative response (4 h treatment), and fatty acid metabolism (4 h treatment) were induced by chitosan (Table 2). Deletion of either *NCU10521*, which encodes a glutathione S-transferase involved in ROS detoxification, or *NCU07840*, which encodes a plasma membrane protein, resulted in increased chitosan susceptibility, which is consistent with the transcriptomic profile [190]. Furthermore, genes associated with the cytoskeleton, cell wall cortex, and vesicle organization were inhibited in response to chitosan (Table 2) [190]. Interestingly, chitosan significantly induced protein synthesis in contrast to the observation in chitosan-treated *A. ochraceus* [189]. These data suggest that the mode of action of chitosan is greatly dependent on the type of chitosan, the properties of chitosan and the particular fungus.

Interestingly, a recent article showed the potential mechanisms of how a fungus is more resistant to chitosan [191]. *Pochonia chlamydosporia* is a nematophagous fungus that can be utilized as a biocontrol against the root-knot nematode *Meloidogyne javanica* [192]. Chitosan not only promotes *P. chlamydosporia* growth [193] but also improves tomato root colonization by *P. chlamydosporia* [194]. Furthermore, chitosan in combination with this fungus reduces damage caused by root-knot nematodes [194]. The greater resistance of *P. chlamydosporia* to chitosan could be due to two mechanisms: (1) The genome of *P. chlamydosporia* contains more chitosanase genes [195], thereby utilizing chitosan as a nutrient source [196]. (2) Many monosaccharide transport genes of *P. chlamydosporia* were induced to assimilate chitosan monomers after chitosan was taken up and degraded into monosaccharides. These findings further demonstrate that the antimicrobial activity of chitosan varies among different microorganisms.

**Figure 2:**
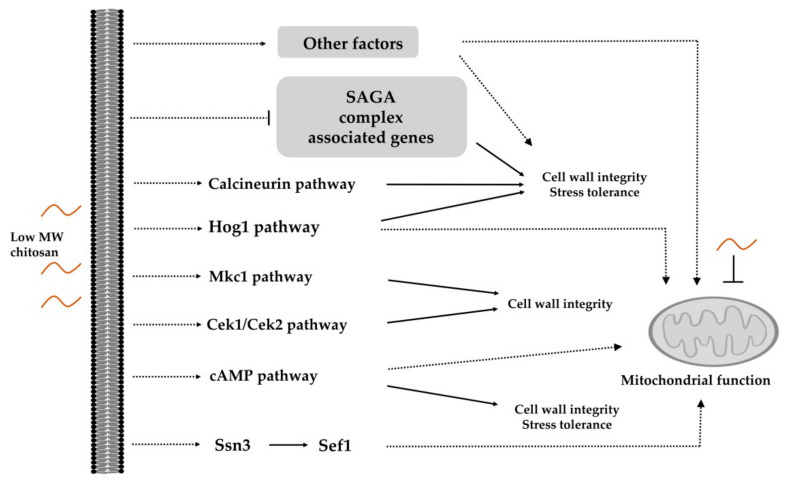
Potential signaling cascades and factors required for chitosan tolerance in *C. albicans*. Mutant library screening has revealed that the Hog1, Cek1/Cek2, Mkc1, Ras1-cAMP, and calcineurin cascades as well as the Ssn3-mediated Sef1 pathway and other factors are involved in chitosan resistance. The roles of these pathways and factors in mediating cell wall integrity might affect mitochondrial function. Furthermore, low-MW chitosan directly represses mitochondrial function, leading to ATP production inhibition.

*C. albicans* is the most frequently isolated fungal pathogen in humans [197,198]. Investigation of the mechanisms of chitosan against *C. albicans* was conducted via mutant library screening [82,123]. These studies identified several genes potentially involved in chitosan resistance (Figure 2). The functions of these genes include adherence, antifungal-related responses, cell surface integrity, stress adaptation, mitochondrial biogenesis, and virulence-associated functions [82,123]. Furthermore, several signaling pathways, such as the Hog1, Cek1/Cek2, Mkc1, Ras1-cAMP, and calcineurin cascades, were proposed to be associated with chitosan tolerance [123]. In particular, chitosan treatment significantly reduced *C. albicans* cell wall thickness via inhibition of the expression of the Spt-Ada-Gcn5-acetyltransferase (SAGA) complex [82]. Furthermore, chitosan represses mitochondrial function by inhibiting *MSS2* [123], which contradicts that observed in *N. crassa* during the response to chitosan [190]. Finally, several calcineurin components and Crz1 TFs were identified during library screening [82,123]. *CRZ1*- and calcineurin-associated deletion strains exhibited high sensitivity to both chitosan and high CaCl_2_ concentrations (unpublished data), suggesting that calcium homeostasis might be associated with chitosan susceptibility. Indeed, in *N. crassa*, the application of exogenous Ca^2+^ could minimize damage caused by chitosan [190].

## 5. Problems Associated with Chitosan

Despite the potential uses of chitosan against microbial infections, there are several concerning issues regarding its properties that may hinder its application: (1) Molecular weight: Chitosan does not have a defined molecular weight, and the molecular weight distribution of each chitosan increases the application difficulty of passing regulatory rules, particularly in the medical field. (2) Purity: Chitosan is made from deacetylated chitin. In general, chitosan with a higher DDA exhibits stronger antimicrobial activity. However, even after treatment with NaOH for a long time and incubation at a high temperature, chitosan with a high DDA (>90%) is produced, indicating that there is less than 10% N-acetylglucosamine in the sample [4,5]. The purity of chitosan might be an issue for application, given that the small amount of N-acetylglucosamine product might affect bioactivity against microbes. (3) Solubility: Chitosan has extremely low solubility under neutral or alkaline pH conditions, and it is dissolved only in acidic environmental conditions [36,68,90,103,107,121], which limits its applications in many areas. Furthermore, a low pH results in more positive charges on chitosan, leading to stronger antimicrobial properties. However, low pH conditions may harm cells, tissues, or organs of the human body.

## 6. Applications of Chitosan-Based Nanoparticles and Films in Combination with Clinical Drugs against Microbes

Chitosan has been widely applied in many areas. However, the antimicrobial effects of pure chitosan and most of its derivatives are still remarkably lower than those of clinical antimicrobial drugs. Several articles have also shown that pure chitosan in combination with clinical drugs exhibits great antimicrobial activity [83,152,199,200,201,202,203]. Thus, this section focuses on the antimicrobial effects of developed chitosan-based biomaterials with current antibacterial and antifungal drugs because chitosan not only has intrinsic antimicrobial properties but is also able to deliver extrinsic antimicrobial drugs (Table 3).

### 6.1. Nanoparticles

Chitosan nanoparticles are synthesized for different purposes by various methods, such as ionotropic gelation, polyelectrolyte complexation, emulsification solvent diffusion, microemulsion, and reverse micelle formation [204,205]. Moreover, the antimicrobial effects of chitosan-based nanoparticles for drug and drug-free delivery systems have been intensively investigated against bacteria and fungi [56,57,58,60,63,67,68,106,119,203,206,207,208,209,210,211,212]. For example, chitosans of different DDAs and MWs exhibited synergistic activity with sulfamethoxazole against *P. aeruginosa* [213]. Chitosan nanoparticles loaded with levofloxacin or clarithromycin showed great potential against methicillin-resistant *S. aureus* (MASA) [211,214]. Additionally, chitosan-silver nanoparticles containing an antibiotic exhibited synergistic effects against fish bacteria [210,211].

### 6.2. Films

PEG-chitosan hydrogels containing ciprofloxacin improved the growth inhibition of *E. coli* compared with drug-free hydrogels and sustainably released the antibiotic for 24 hr [70]. Similarly, the high DDA of chitosan films loaded with different antibiotics exhibited better activity against different pathogenic bacteria [55,215,216,217,218]. Fibrin-chitosan loaded with two antibiotics (metroidazole and ciprofloxacin) enhanced anti-*Enterococcus faecalis* activity [78]. A chitosan hydrogel containing ciprofloxacin and fluconazole nanoparticles exhibited significant antimicrobial activities against *C. albicans*, *E. coli*, and *S. aureus* [219]. Finally, a chitosan gel with metronidazole showed great anti-*Candida* activity to treat vaginal infection [220].

### 6.3. Implants

Chitosan-coated titanium containing tetracycline or chlorhexidine digluconate effectively inhibited *Actinobacillus actinomycetemcomitans* and *Staphylococcus epidermidis* [221]. Interestingly, a chitosan bar containing gentamicin prepared using crosslinking, solvent evaporation, and a cylinder model cutting technique, which was implanted into rabbit tibias, exhibited significant antibacterial activity, suggesting that this chitosan bar would be effective against chronic osteomyelitis [222].

## 7. Conclusions

Approximately 30,000 original research and review articles related to chitosan have been reported [223], indicating that this naturally occurring product has great potential applications. This review suggests that chitosan as a natural antimicrobial agent can be applied in agriculture, food, and biomedical areas. Transcriptomic analyses in chitosan-treated microbes have further concluded that the mode of action of chitosan against bacteria or fungi may have multiple intracellular and extracellular effects. Although chitosan shows great promising antimicrobial potential, most of these studies are still at the laboratory level. Furthermore, the low water solubility and the lack of defined molecular weight and purity are the major issues for future application of chitosan. The development of better strategies and optimized conditions against pathogenic bacteria and fungi is necessary.

## Figures and Tables

**Table 1:** Genetic responses of chitosan-treated bacteria.

Bacteria	Functional Categories		Conclusions	Ref.
*S. aureus*	Upregulation	**84 genes** Membrane Bioenergetics Cell Division Metabolism of Carbohydrates Metabolism of Amino Acids Regulation of RNA Synthesis Protein Folding Adaptation to Atypical Conditions Phage-Related Functions	Chitosan binds to teichoic acids Chitosan increases membrane permeability and causes membrane depolarization Chitosan might be involved in energy metabolism	[107]
	Downregulation	**82 genes** Transport/Binding Proteins and Lipoproteins Metabolism of Nucleotides and Nucleic Acids Metabolism of Lipids		
*B. cereus*	Upregulation	**57 genes** Potassium Transport System Membrane Protein-Associated Genes	Chitosan caused the permeabilizing effect, resulting in the leakage of intracellular potassium Deletion of the *kdp* gene (an ATP-driven K^+^ transport system) exhibited no significant difference in the minimum inhibitory concentration (MIC) and minimum bactericidal concentration (MBC)	[185]
	Downregulation	**51 genes** Chitin Binding Protein Metabolism of Amino Acids and Other Cellular Constituents Gluconeogenesis		

**Table 2:** Genetic responses of chitosan-treated fungi.

Fungus	Functional Categories		Conclusion	Ref.
*S. cerevisiae*	Upregulation	**Treated for 15 min/46 genes** ER Integral to Membrane **Treated for 30 min/97 genes** ER Integral to Membrane Cell Wall Cell Wall Organization and Biogenesis Vacuoles Plasma Membrane **Treated for 60 min/97 genes** ER Integral to Membrane Cell Wall Cell Wall Organization and Biogenesis Vacuoles **Treated for 120 min/234 genes** ER Integral to Membrane Cell Wall Cell Wall Organization and Biogenesis Vacuoles Stress Response **Treated 180 min/432 genes** ER Integral to Membrane Cell Wall Cell Wall Organization and Biogenesis Vacuoles Response to Stress	Chitosan may be representative of other plasma membrane-perturbing compounds Chitosan stress decreases translational activity Calcineurin-dependent pathway is involved Deletion of *CIN5*, *CRZ1*, or *RIM1* exhibits high sensitivity to chitosan	[186]
	Downregulation	**Not available** rRNA Processing Ribosomes		
*S. cerevisiae*	Upregulation	**589 genes** Transcription Cell Cycle Protein Modification Stress Response RAS Signal Transduction	COS does not have specific gene targets Membrane permeability is increased in the COS-treated budding yeast Synergistic antifungal activity between chitosan and fluconazole was found	[187]
	Downregulation	**631 genes** Protein Folding Protein Complex Assembly Mitochondrial Electron Transport		
*A. ochraceus*	Upregulation	**309 genes** Starch and Sucrose Metabolism Glycerophospholipid Metabolism Ether Lipid Metabolism Steroid Biosynthesis Mitochondrial Electron Transport	Chitosan damages the integrity of the cell surface architecture and affects membrane fluidity Chitosan affects protein biosynthesis Chitosan is an alternative compound to control fungal pathogens	[189]
	Downregulation	**26 genes** Ribosome Biogenesis in Eukaryotes Glycerophospholipid Metabolism Ether Lipid Metabolism Steroid Biosynthesis		
*N. crassa*	Upregulation	**237 genes in total (4, 8, and 16 h of treatment)** Peroxisome Organization, ROS Degradation and Fatty Acid Catabolism (4 h) Mitochondrial Function (4 h, 8 h and 16 h) Ribosome and Ribosome Biogenesis (16 h) Nucleolus (8 h and 16 h) Structural Molecule Activity (16 h)	A MFS transporter (NCU04534) and a glutathione transferase (NCU10521) are the targets of chitosan Chitosan treatment causes an increase in intracellular ROS Chitosan affects protein biosynthesis *ΔNCU03639* (lipase), *ΔNCU04537* (monosaccharide transporter), *ΔNCU10521* (glutathione S-transferase), *ΔNCU08907* Clock controller gene 13 (*ccg-13*) and *ΔNCU07840* (plasma membrane protein with a *het* domain) are sensitive to chitosan The presence of Ca^2+^ increases chitosan tolerance	[190]
	Downregulation	**291 genes in total (4, 8, and 16 h treatment)** Peroxisome Organization, ROS Degradation and Fatty Acid Catabolism (16 h) Cell Cortex (4, 8, and 16 h) Vesicle Organization (8 and 16 h) Conjugation (4, 8, and 16 h) G Protein Receptor Signaling Pathway (16 h) Microtubule Organizing Center (4, 8, and 16 h) Ribosome and Ribosome Biogenesis (4 h) Nucleolus (4 h) Structural Molecule Activity (4 h)		
*P. chlamydosporia*	Upregulation	**46 genes** Redox Processes Carbohydrate Catabolism Proteolysis Carbohydrate Transport Cell Cycle Energetic Metabolism Lipid Metabolism Protein Synthesis and Modification Chitin and Chitosan Degradation Structural Constituent of Cell Wall	Chitosan activates the expression of cytochrome P450 ClCP1 and thioredoxin-like proteins *P. chlamydosporia* is more resistance to chitosan because it may contain more chitosanase genes Chitosan induces many monosaccharide transport genes Chitosan could be a non-toxic additive to reduce root-knot nematode parasitism	[191]
	Downregulation	**90 Genes** Oxidation–Reduction Metabolism Cellular Protein Metabolic Process Macromolecule Biosynthetic Process Small Molecule Metabolic Process Metal Transport		

**Table 3:** Applications of chitosan-based biomaterials containing clinical antimicrobial drugs.

Chitosan/Antimicrobial Drug	Chitosan-Based Biomaterial	Findings	Microorganism(s)	Ref.
Chitosan/Sulfamethoxazole	Nanoparticle	Synergistic activity with sulfamethoxazole	*P. aeruginosa*	[213]
Chitosan/Amoxicillin Cefixime Levofloxacin	Nanoparticle	Significant antibacterial activity	*P. aeruginosa* *E. coli* *S. aureus* *Salmonella typhi* *Klebsiella pneumoniae*	[211]
Chitosan/Amikacin Rifampicin	Nanoparticle	Antibacterial activity against resistant strains	*Aeromonas hydrophila* *Edwardsiella tarda* *Pasteurella piscicida* *P. aeruginosa* *Streptococcus faecium* *Streptococcus iniae* *Vibrio ordalli* *Yersinia ruckeri*	[210]
Chitosan/Ciprofloxacin Chlortetracycline Hydrochloride Gentamycin sulfate	Nanoparticle	Inhibits the growth of gram-positive and gram-negative bacteria	*E. coli* *S. aureus*	[57]
Chitosan/Azithromycin Levofloxacin Tetracycline	Nanoparticle	Shows significant antibacterial effects	*E. coli* *S. aureus*	[203]
Chitosan/Rifampicin Ciprofloxacin Vancomycin Doxycycline Gentamicin	Nanoparticle	Inhibits bacterial biofilm and exhibits synergism with antibiotics	*S. epidermidis*	[206]
Chitosan/Clarithromycin	Nanoparticle	Shows antibacterial activity	*S. aureus*	[212]
Chitosan/Ciprofloxacin	Hydrogel	Inhibits bacterial growth	*E. coli*	[70]
Chitosan/Clindamycin	Hydrogel	Enhances the antibacterial properties	*E. faecalis*	[78]
Chitosan/Ciprofloxacin Fluconazole	Hydrogel(Bandage)	Shows significant antimicrobial activity	*C. albicans* *E. coli* *S. aureus*	[219]
Chitosan/Minocycline Rifampicin	Hydrogel	Provides bactericidal activity directly to the wound site	*E. coli* *S. aureus*	[217]
Chitosan/Tetracycline	Hydrogel	Has potential applications for antimicrobial action	*S. aureus*	[55]
Chitosan/Amikacin Daptomycin	Film	Effectively inhibits the growth of bacteria	*S. aureus*	[218]
Chitosan/Daptomycin Vancomycin	Film	Shows activity against gram-positive bacteria	*S. aureus*	[215]
Chitosan/Clotrimazole	Solid Mixtures	Acts synergistically with clotrimazole against non-*albicans Candida* strains	*Candida glabrata*	[216]
Chitosan/Levofloxacin	Hydroxypropyl methyl cellulose (HPMC) gel	Antibacterial activity against resistant strains	*Methicillin-resistant* *S. aureus*	[214]
Chitosan/Metronidazole	HPMC Gel	Anti-*Candida* activity	*Candida species*	[220]
Chitosan/Tetracycline Chlorhexidine	Chitosan-Coated Titanium Pins	Inhibits pathogen growth	*A. actinomycetemcomitans* *S. epidermidis*	[221]
Chitosan/Gentamicin	Chitosan Bar	Shows significant antibacterial activity	Microbes	[222]
